# The Immune Mechanisms of Lung Parenchymal Damage in Tuberculosis and the Role of Host-Directed Therapy

**DOI:** 10.3389/fmicb.2018.02603

**Published:** 2018-10-30

**Authors:** Cari Stek, Brian Allwood, Naomi F. Walker, Robert J. Wilkinson, Lutgarde Lynen, Graeme Meintjes

**Affiliations:** ^1^Wellcome Centre for Infectious Diseases Research in Africa, Institute of Infectious Disease and Molecular Medicine, University of Cape Town, Cape Town, South Africa; ^2^Department of Clinical Sciences, Institute of Tropical Medicine Antwerp, Antwerp, Belgium; ^3^Department of Medicine, University of Cape Town, Cape Town, South Africa; ^4^Division of Pulmonology, Department of Medicine, Stellenbosch University, Stellenbosch, South Africa; ^5^Department of Clinical Research, London School of Hygiene & Tropical Medicine, London, United Kingdom; ^6^Department of Medicine, Imperial College London, London, United Kingdom; ^7^Francis Crick Institute, London, United Kingdom

**Keywords:** tuberculosis, lung damage, host-directed therapy, cavity, pulmonary function, matrix metalloproteinase, neutrophils, immune mechanisms

## Abstract

Impaired lung function is common in people with a history of tuberculosis. Host-directed therapy added to tuberculosis treatment may reduce lung damage and result in improved lung function. An understanding of the pathogenesis of pulmonary damage in TB is fundamental to successfully predicting which interventions could be beneficial. In this review, we describe the different features of TB immunopathology that lead to impaired lung function, namely cavities, bronchiectasis, and fibrosis. We discuss the immunological processes that cause lung damage, focusing on studies performed in humans, and using chest radiograph abnormalities as a marker for pulmonary damage. We highlight the roles of matrix metalloproteinases, neutrophils, eicosanoids and cytokines, like tumor necrosis factor-α and interleukin 1β, as well as the role of HIV co-infection. Finally, we focus on various existing drugs that affect one or more of the immunological mediators of lung damage and could therefore play a role as host-directed therapy.

## Introduction

In 2016, an estimated 10.4 million people developed tuberculosis (TB) worldwide. Although effective diagnosis and treatment saved about 53 million lives between 2000 and 2016, TB remains a major threat worldwide: 16% of TB cases die from the disease, corresponding to 1.7 million deaths in 2016 (World Health Organization, 2017). Among those who are cured successfully, residual pulmonary impairment is common. Various studies have looked at lung function in patients with a known history of TB; they found abnormal lung function in 34 – 94% of patients, varying in severity from mild to severe (Willcox and Ferguson, 1989; Plit et al., 1998; de Valliere and Barker, 2004; Chung et al., 2011; Vecino et al., 2011; Akkara et al., 2013; Baez-Saldana et al., 2013; Ralph et al., 2013a; de la Mora et al., 2015; Nihues Sde et al., 2015; Manji et al., 2016). It results in considerable medical costs (Jordan et al., 2010) and decreased quality of life (Ralph et al., 2013a; de la Mora et al., 2015).

Impaired lung function is associated with chest radiograph (CXR) abnormalities in most of the studies. It can easily be measured using spirometry, which measures air volumes and airflow rates of the lung. Forced vital capacity (FVC) is the maximal volume of air exhaled by a patient from the position of maximal inspiration, by means of a rapid, maximally forced expiration; forced expiratory volume in 1 s (FEV1) is the amount of air exhaled during the first second of the FVC maneuver. The nature and severity of pulmonary impairment can be categorized by combining these two measurements: obstruction is defined as a FEV1/FVC ratio < 70%, restriction is suggested by a low FVC (<80% of the predicted value). Obstruction, low FVC, and mixed defects have all been reported in patients with previous TB.

## Purpose of Review

The aim of TB treatment is to kill the causative mycobacteria with anti-mycobacterial agents. Because of the lengthy duration of the treatment, the possibilities of drug toxicity, and increasing drug resistance, host-directed therapies (HDT), have gained attention (Hawn et al., 2013; Wallis and Hafner, 2015; Zumla et al., 2015). HDTs are agents that can augment host defense mechanisms, modulate excessive inflammation or both, by manipulating the hosts response to a pathogen rather than targeting the pathogen itself. This may lead to improved clinical treatment outcomes such as reduced morbidity, mortality, and end-organ damage, and long-term functional recovery. Supplementing anti-TB treatment with drugs that reduce pulmonary damage could result in improved pulmonary function. To predict which interventions could be beneficial, an understanding of the pathogenesis of pulmonary damage in TB is important. What are the immunological processes leading to lung damage in humans? Where and how in the process could we intervene to prevent or reduce lung damage? How much damage is already done at diagnosis and how much still occurs during treatment?

## What Does Pulmonary Damage in Human TB Look Like?

The established paradigm positions the caseating granuloma as the characteristic lesion of TB. However, this paradigm originates from animal studies in the late 20th century, when data on histology of human TB had become rare. Studies done before the 1950s describe two characteristic presentations in human pulmonary TB: the caseous granuloma and the tuberculous pneumonia. They divide lung pathology into primary and post-primary TB. Primary TB is the infection that occurs when people first encounter *Mycobacterium tuberculosis (Mtb).* Post-primary TB occurs later, as a result of reactivation of latent TB or reinfection, and causes the majority of clinical TB (Hunter, 2011). The two differ with regard to their location in the lung, the host immune response and their histopathology. Primary TB typically occurs mainly in the lower zones of the lung. It is usually self-limiting but leads to consolidative pneumonia or lymphadenitis in a small proportion of individuals. It is characterized by a greater bacillary load and reduced lipid accumulation in the alveoli and the interstitium compared to post-primary TB, as well as an acute inflammatory response; cavitation however, is rare. Post-primary TB is said to develop mainly in the apices of the lung. It is characterized by obstructive pneumonia, which is frequently asymptomatic in its early stages. Endobronchial spread from the small peripheral airways can lead to necrotic caseous pneumonia, associated with progressive tissue necrosis and cavity formation or fibrocaseous disease (Long et al., 1998; Hunter, 2016). TB typically heals with persisting cavities, scarring, and pleural adhesions, as observed in autopsies of persons with previous TB who died of other causes (Theegarten et al., 2006). However, abnormal findings need not be present and viable TB can be found in both macroscopically normal and abnormal appearing lung tissue (Kuhne and Willgeroth, 1988).

Chest radiographs are commonly used to visualize pulmonary damage. Radiologists distinguish primary and post-primary TB as the two typical patterns in active TB. Primary TB is characterized by lymphadenopathy and air space consolidation often in the middle or lower lobes, with or without an accompanying pleural effusion. Post-primary TB consists of consolidation and/or nodules, frequently in the upper lobes or apices of the lower lobes, with or without cavitation (Nachiappan et al., 2017). CXRs of people with previous TB show abnormalities in 14–100%, including fibrosis, bronchiectasis, and persisting cavities, the latter occurring more often in re-treatment patients or those with multi-drug resistant TB (Meghji et al., 2016). All these abnormalities are associated with impaired lung function.

Computed tomography (CT) scans are more sensitive than CXRs, especially for imaging of centrilobular small nodules or the so-called tree-in-bud sign; these classical features of early endobronchial spread of TB are often underestimated on a CXR (Skoura et al., 2015); [^18^F]-fluoro-2-deoxy-D-glucose positron emission tomography (FDG-PET) with CT combines anatomic imaging with imaging of metabolic activity of lesions. It has been used in TB to follow the evolution of lung lesions during treatment (Martinez et al., 2012; Malherbe et al., 2016) and, importantly, has shown that metabolically active lung lesions may be present before the onset of clinical disease (Esmail et al., 2016), and persist after treatment completion (Malherbe et al., 2016).

## What Happens After Mtb Enters the Lung?

After *Mtb* enters the lung, the bacilli are taken up by alveolar macrophages, dendritic cells, and neutrophils, or occasionally epithelial cells; the latter possibly resulting in limited early bacterial growth. Infected cells start producing and secreting antimicrobial peptides, cytokines (like interleukin (IL)-1β, tumor necrosis factor (TNF)-α, IL-12, and IL-6) and chemokines. Other immune cells and permissive macrophages are attracted to the site of infection (O’Garra et al., 2013). *Mtb* itself, using multiple strategies, directs the recruitment of macrophages and triggers granuloma formation (Ndlovu and Marakalala, 2016). Secondary granulomas are formed by infected macrophages departing the primary granuloma or when a granuloma ruptures. While *Mtb* replicates freely in the macrophages, dendritic cells migrate to the local lymph nodes, to activate T cells. The arrival of *Mtb* specific T-cells in the lung usually does not happen until 14–21 days after initiation of the infection (Gallegos et al., 2008). Their production of TNF-α and interferon-γ (IFN-γ) stimulates killing activities by macrophages. Moreover, T-cells complete granuloma formation by forming the lymphocytic cuff surrounding it (O’Garra et al., 2013).

The balance between the eicosanoids prostaglandin E2 (PGE2) and lipoxin A4 (LXA4) affects the mode of death of infected macrophages. LXA4 promotes macrophage necrosis, resulting in cell lysis of the macrophage, thereby allowing *Mtb* to escape and spread to neighboring cells. PGE2 stimulates apoptosis, leaving the macrophage plasma membrane intact, containing the bacilli, and enhancing immunity (Chen et al., 2008). Leukotriene (LT) B4, through regulation of TNF-α production (Tobin et al., 2012) and possibly attraction of neutrophils (Lammermann et al., 2013), is also involved, with both high and low levels of LTB4 inducing macrophage necrosis (Tobin et al., 2012).

In only 10% of individuals, progressive primary disease occurs; in the remaining 90% the initial infection is contained and latent infection is established (O’Garra et al., 2013). Current thinking views active and latent TB on a spectrum of tuberculosis disease, rather than as two distinct disease states as historically classified. (Barry et al., 2009).

## Granulomas

Most human granulomas are composed of a center of infected macrophages, with the ability to differentiate, for example into epithelioid cells, multi-nucleated giant cells, and foamy macrophages. An outer layer of lymphocytes surrounds these cells, and many other cells, including neutrophils, dendritic cells, natural killer (NK) cells and fibroblasts may form part of the granuloma. The granuloma contains the mycobacteria, preventing their spread, but at the same time serves as a site of replication and persistence for *Mtb* (Ndlovu and Marakalala, 2016). Different types of granuloma exist: cellular, suppurative, fibrotic, or caseous (Canetti, 1955). Caseous necrosis occurs when cells within the granuloma undergo necrosis (O’Garra et al., 2013); alternatively, it has been suggested that – in post-primary TB - granulomas form in response to existing areas of necrotic caseous pneumonia (Hunter, 2016). Caseous necrosis happens in conjunction with extracellular matrix (ECM) destruction. In the classical paradigm, tissue destruction occurs as a result of caseous necrosis (O’Garra et al., 2013). However, an alternative theory proposes that collagen destruction precedes caseation and, therefore, ECM destruction is the initial pathological event (Al Shammari et al., 2015).

Diverse types of granulomas can be present in one lung at the same time, ranging from small cellular granulomas to multiple caseous granulomas that coalesce and expel their contents to form large cavities; they behave independently of each other, and different immunologic profiles exist between (Ulrichs et al., 2005; Subbian et al., 2015) and within (Marakalala et al., 2016) granulomas. Granulomas can be stable, or either resolve or progress. Clinically, the behavior of a few or even a single poorly controlled granuloma can determine the outcome of the disease on a host level (Flynn, 2018).

## Cavities, Bronchiectasis and Fibrosis

The lung consists of both cellular and extracellular components. The ECM is comprised of the interstitial connective tissue matrix, which forms the parenchyma of the lung, surrounding cells and providing structural scaffolding, and the basement membrane, which separates the alveolar epithelium or endothelium from the surrounding stroma. Support of the alveoli by the ECM is needed for normal lung function; destruction or abnormal remodeling of the ECM occurs in many pulmonary diseases and leads to pulmonary impairment (Elkington and Friedland, 2006). The ECM of the lung is mainly made up of type I collagen and elastin. Type III and IV collagen are important components of the alveolar wall and basement membrane. Large fibers are connected by smaller fibrils. Dissemination of mycobacteria from the lung parenchyma into the airways as well as formation of cavities requires destruction of the ECM through cleavage of both small fibrils and large fibers. Collagens, however, are highly resistant to cleavage by proteolytic enzymes; only matrix metalloproteinases (MMPs) are capable of completely degrading the ECM (Elkington and Friedland, 2006). Consequently, MMPs play an important role in the development of cavities, bronchiectasis as well as fibrosis.

The development of cavities in TB has been studied extensively in rabbits, using *Mycobacterium bovis*. In these studies, cavities developed from liquefied caseating granulomas, that contained large numbers of actively growing bacteria. Bacteria release high amounts of tuberculin-like products causing a tissue-damaging delayed-type hypersensitivity reaction (Dannenberg, 2006). This T-cell mediated immune reaction is important; cavities developed mainly in pre-sensitized rabbits and desensitization or immune suppression could prevent cavity formation (Yamamura et al., 1968; Yamamura et al., 1974). Cavities are formed when expanding granulomas ruptures their caseous contents into a bronchus (Dannenberg, 2006).

Histologic studies in humans show a different picture of cavity formation that challenges the paradigm described in rabbits (Hunter, 2016): cavities do not develop from liquefied caseating granulomas, but from a caseous pneumonia. Host lipids and mycobacterial antigens accumulate in the alveoli, but only small numbers of bacteria are present. Similar to the rabbit model, sudden necrosis related to a delayed-type hypersensitivity reaction against mycobacterial antigens occurs (Hunter, 2016). However, an alternative yet controversial theory, based on the small numbers of bacteria observed and several observations related to autoimmunity seen in patients with TB, proposes a role for autoimmunity: mycobacteria induce inappropriate host responses to self-antigens, causing autoimmune inflammation (Elkington et al., 2016). A considerable overlap in gene expression signatures between TB and autoimmune diseases, greater than seen with other infectious diseases, supports this theory (Clayton et al., 2017).

The lipid-rich necrotic material in granulomas does not have the enzymatic activity to degrade collagen and consequently, its build-up is only one component of cavity formation. Extracellular matrix breakdown takes place and involves MMPs. Indeed, increased concentrations of MMPs have been found in TB cavities in rabbits (Kubler et al., 2015) and in humans (Sakamoto et al., 2013; Ong et al., 2015). Neutrophils have also been found in cavities (Ong et al., 2015).

Bronchiectasis, an irreversible dilatation of the bronchi, is caused by an ongoing inflammatory process (like TB), which results in damage to the airway epithelium, leading to an inability to clear secretions, as well as destruction of the elastin in the airway walls (Milliron et al., 2015). Similar to cavity formation, MMPs have been implicated in the development of bronchiectasis, with increased levels being found in sputum, bronchoalveolar lavage fluid (BALF), and the lamina propria of patients with bronchiectasis (Sepper et al., 1995; Zheng et al., 2002; Guan et al., 2015). Neutrophils, together with macrophages and T-cells, are the dominant cell type in bronchiectatic inflammation (King, 2009). Alternatively, traction bronchiectasis can occur, secondary to scarring of the adjacent parenchyma or narrowing of more proximal bronchi (Milliron et al., 2015).

Fibrosis results from the excessive deposition of components of the ECM such as collagen and fibronectin in and around inflamed or damaged tissue by myofibroblasts. Its pathogenesis is complicated (Wynn and Ramalingam, 2012), with many innate and adaptive immune cells and cytokines playing a role. Transforming growth factor (TGF-β), produced by macrophages, lung epithelial cells, and fibroblasts, is one of the key players (Wynn and Ramalingam, 2012) and indeed, higher levels of TGF-β in serum and BALF correlate with an increase in fibrosis seen on high-resolution CT scan in patients with TB 6 months after the start of treatment (Ameglio et al., 2005). TNF-α, IL-β, and IL-17-induced neutrophil recruitment also seems to play a crucial role in the development of fibrosis (Wynn and Ramalingam, 2012). MMPs appear to be involved: some MMPs reduce fibrosis, but others – perhaps counterintuitively – promote it (Giannandrea and Parks, 2014). In a Taiwanese study, patients with an *MMP-1 (-1607G)* gene polymorphism, leading to excessive MMP-1 production, were more likely to have moderate to advanced fibrosis on CXR 1 year after completion of TB treatment (Wang et al., 2010).

## What Are the Immunological Mediators and Processes Leading to Lung Damage?

Much of our recent knowledge of immunological processes in TB comes from animal models. Mice, rabbits, guinea pigs, and zebra-fish have all been used to study TB. However, none of these models completely replicate the immunopathology seen in human TB. More recently, non-human primates have also been used, exhibiting a spectrum of pathology closely resembling TB in humans (Flynn et al., 2015).

For this review, we included studies done in humans, where serum and BALF markers are commonly used to assess the immunological processes in the lung. Serum measurements reflect systemic responses and do not represent what happens in individual granulomas, as was shown by a difference in gene expression patterns between granuloma and blood (Subbian et al., 2015). BALF more closely reflects responses taking place in the lung, however, even BALF only reflects processes taking place in the airways and not necessarily those in the lung parenchyma. Histology is the only way to assess the immunological processes occurring within a granuloma; however, histological samples are more difficult to obtain and, therefore, most study findings in humans are built on assumptions using available body fluid. Studies that do include histological samples cannot present longitudinal data.

**FIGURE 1:**
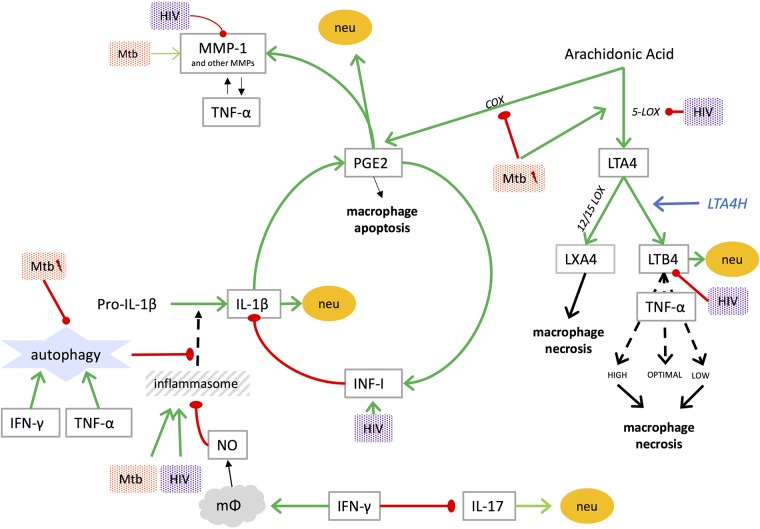
Mediators of lung damage in TB and interplay with [lightning flash cartoon], virulent *Mtb;* COX, cyclooxygenase; IFN-I, type I interferon; IFN-γ, interferon gamma; IL, interleukin; LOX, lipoxygenase; LT, leukotriene; LTA4H, leukotriene A4 hydrolase; LX, lipoxin; mφ, macrophage; MMP, matrix metalloproteinase; Mtb, mycobacterium tuberculosis; neu, neutrophil recruitment; NO, nitric oxide; PGE2, prostaglandin E2; TNF, tumor necrosis factor. NO inhibits assembly of the NLRP3 inflammasome (Mishra et al., 2013).

When conducting our review, we searched for studies that assessed inflammatory mediators, and associated them with radiological abnormalities as a marker for pulmonary damage (Figure 1).

## Matrix Metalloproteinases

There are 23 MMPs in humans. They can be secreted by a variety of cells, including macrophages/monocytes, neutrophils, and lung epithelial cells. Their generation is tightly regulated. They are not stored requiring gene transcription immediately before secretion; exceptions being MMP-8 and -9 stored in neutrophils. Once activated, they are regulated by endogenous inhibitors, called tissue inhibitors of metalloproteinases (TIMPs). Expression of MMPs is increased by prostaglandin and several cytokines (including IL-1β, IL-17 (Singh et al., 2018), TNF-α, and IFN-γ) (Elkington P.T. et al., 2011); hypoxic conditions, present in TB lesions, also increase expression and secretion of MMP-1 through the induction of hypoxia-inducible factor 1α (Belton et al., 2016). A recent study has demonstrated a role for platelets in MMP-1 upregulation in *Mtb-*infected monocytes, in addition to upregulation of IL-1β and IL-10 (Fox et al., 2018).

As described above, degradation of collagens and elastins by MMPs during active TB leads to the formation of cavities. Strong evidence of the role of MMPs in lung damage comes from studies in transgenic mice expressing human MMP-1. Wildtype mice do not express the ortholog of MMP-1 in lung and do not develop caseous necrosis or cavities in response to *Mtb*; in human MMP-1 transgenic mice, however, infection with TB leads to collagen destruction and caseous necrosis (Elkington P. et al., 2011; Al Shammari et al., 2015). MMPs also play a role in granuloma formation (Parasa et al., 2017).

Several MMPs are upregulated in blood, sputum, and BALF of patients with active TB, primarily MMP-1, -3, -7, -8, and -9 (Elkington P.T. et al., 2011). MMP-1 is the dominant collagenase in TB (Elkington P. et al., 2011); its secretion is driven by *Mtb* directly by activation of multiple intracellular signaling pathways and by intercellular networks (Ong et al., 2014). Corresponding TIMPs are not similarly upregulated by *Mtb*, leading to a matrix-degrading phenotype in TB (Price et al., 2001). In a zebrafish model, using *M. marinum* to study granuloma formation, mycobacterial-derived ESAT-6 induced MMP-9 secretion, enhancing monocyte recruitment to granulomas (Taylor et al., 2006; Volkman et al., 2010).

Increased levels of MMPs correlate with pulmonary damage: sputum levels of MMP-1, -2, and -8 were elevated in patients with cavities and correlated positively with the extent of infiltrates on CXR (Walker et al., 2012; Ong et al., 2015). Similarly, sputum levels of membrane type-1 MMP (a membrane-bound collagenase expressed on monocytes), plasma concentrations of procollagen III N-terminal propeptide (PIIINP, a degradation product of collagen type III), BALF levels of MMP-3, -7, and -8, and serum concentrations of MMP- 1, -8, and -9, correlated with more extensive CXR abnormalities in patients with TB from several different countries (Hrabec et al., 2002; Seddon et al., 2013; Singh et al., 2014a; Sathyamoorthy et al., 2015; Sigal et al., 2017). These findings suggest a central role for MMPs and extracellular matrix degradation in the development of lung damage in TB.

## Neutrophils

Neutrophils are abundant in the airways of humans with active TB (Eum et al., 2010). Their role in TB appears dichotomous: high numbers of neutrophils in the blood at the time of exposure are associated with lower likelihood of infection (Martineau et al., 2007). Conversely later in TB their numbers in blood were associated with worse patient outcomes (Barnes et al., 1988; Lowe et al., 2013). Various soluble mediators (amongst others Il-1β, IL-8, IL-17, PGE2, LTB4, and granulocyte colony-stimulating factor) promote neutrophil recruitment (Lowe et al., 2012); others, like IFN-γ and nitric oxide (NO), reduce neutrophil recruitment and survival, partly via inhibition of IL-17 (Nandi and Behar, 2011), IL-1β and 12-lipooxygenase (12-LOX) (Mishra et al., 2017).

At the time of presentation with active TB, neutrophils are associated with lung damage: a neutrophil-driven, IFN-inducible whole-blood transcript signature (Berry et al., 2010), higher blood (Abakay et al., 2015; Panteleev et al., 2017) and BALF (Nolan et al., 2013) neutrophil counts, and higher serum levels of S100 proteins (a protein produced by neutrophils, promoting their own recruitment) (Gopal et al., 2013; Berrocal-Almanza et al., 2016) in patients with active TB all relate with the extent of lung radiographic disease. Lung damage is thought to be contributed to by their indiscriminate killing mechanisms, which can result in significant bystander damage to surrounding host tissue. Moreover, neutrophils are the only cells that store MMPs (Ong et al., 2015), while they do not synthesize TIMPs, thus allowing for unrestrained effects of MMPs (Masure et al., 1991). Removing infected or dying neutrophils is necessary to protect the surrounding tissue. Removal of apoptotic neutrophils by macrophages promotes subsequent killing of *Mtb*, whereas removal of necrotic neutrophils allows for mycobacterial survival and proliferation inside the macrophages. *Mtb* drives neutrophil necrosis, a process that requires neutrophil-derived reactive oxygen species (ROS) (Dallenga et al., 2017b). Inhibition of ROS-production could restore growth control of *Mtb* by macrophages (Dallenga et al., 2017a).

## Eicosanoids

The eicosanoids PGE2, LXA4, and LTB4 are all metabolites of arachidonic acid (AA). Cyclooxygenase (COX) converts AA into PGE2, while 5-lipooxygenase (5-LOX) generates LTA4, which is again converted into either LXA4 by 12-LOX, or LTB4 by leukotriene A4 hydrolase (LTA4H) (Dietzold et al., 2015). As mentioned previously, the balance between these eicosanoids influences the mechanism of macrophage death (Chen et al., 2008). Macrophage apoptosis leads to an early immune response with better control of the infection and minimal immunopathology, while macrophage necrosis leads to a delayed immune response, inadequate control of infection and greater immunopathology (Divangahi et al., 2013). Virulent strains of *Mtb* promote LXA4 production, thereby stimulating necrosis and mycobacterial spread (Chen et al., 2008). To our knowledge, no studies have correlated PGE2 or LXA4 with pulmonary function in human TB; one can speculate that tipping the eicosanoid-balance toward PGE2 may result in less lung damage. Findings in mice and latent TB in humans, however, show that levels of PGE2 were low early in the infection and increased later in and during active TB (Rangel Moreno et al., 2002; Shu et al., 2013; Mayer-Barber et al., 2014; Lee et al., 2015). This underlines the complex and poorly elucidated role of PGE2 in TB infection and may even suggest a changing role for PGE2 during the course of the disease. LTB4, which is generated by LTA4H, has been correlated with severity of TB on CXRs in one study (el-Ahmady et al., 1997).

## Cytokines

Various studies have assessed the association between cytokines (including IFN–y and TNF–α, and several pro- and anti-inflammatory interleukins) and CXR abnormalities in TB (Dlugovitzky et al., 1997; Sodhi et al., 1997; Casarini et al., 1999; Tsao et al., 1999, 2000, 2002; van Crevel et al., 2000; Mazzarella et al., 2003; Ameglio et al., 2005; Wu et al., 2007; Berry et al., 2010; Su et al., 2010; Walker et al., 2012; Nolan et al., 2013; Chowdhury et al., 2014; Fan et al., 2015; Sigal et al., 2017). The different measuring methods used and the fact that several cytokines are not limited to a single effector function make comparison and interpretation challenging.

Only TNF-α and IL-1β in both blood and BALF seem to unambiguously correlate with CXR abnormalities. Higher levels of TNF-α and IL-1β correlate with the presence or size of cavities (Tsao et al., 2000; Ameglio et al., 2005; Chowdhury et al., 2014; Sigal et al., 2017) and with the extent of pulmonary involvement (Casarini et al., 1999; Walker et al., 2012). Moreover, lower levels of these cytokines were found in patients with an early radiological response to TB treatment (improved CXR after 2 months of treatment) compared to those with a later (at 6 months) response (Su et al., 2010). In animal models, the effect of TNF-α seems to be dose dependent, where both high and low doses lead to tissue destruction (Bekker et al., 2000; Tobin et al., 2012). LTA4H polymorphism, and subsequently eicosanoid patterns, play a role in its regulation (Tobin et al., 2012). Both TNF-α and IL-1β affect secretion of MMPs and MMPs in their turn can play a role in the release, activation or inactivation of TNF-α and IL-1β (Elkington and Friedland, 2006). IL-1β also associates with activation of fibroblasts (Borthwick, 2016) and the recruitment of neutrophils (Lowe et al., 2012; Mishra et al., 2017), which all associate with lung damage.

## Autophagy

Autophagy is an intracellular self-digestion process: cytosolic material is engulfed by a double-membrane vesicle called the autophagosome, that delivers it to lysosomes for degradation and subsequently releases the degraded products back to the cytosol. Autophagy can be used by the host to eliminate intracellular pathogens and plays an important role in defense against *Mtb* (Gutierrez et al., 2004); both IFN-γ and TNF-α can induce autophagy (Songane et al., 2012). It can also downregulate IL-1β production mediated through the inflammasome (an intracellular multiprotein complex that triggers formation of proinflammatory cytokines), by removing large inflammasome complexes or damaged mitochondria - which, through production of ROS, trigger the inflammasome (Rathinam et al., 2012). Virulent *Mtb* can inhibit autophagy (Gupta et al., 2016), subsequently leading to increased IL-1β production (Songane et al., 2012). It was found that patients infected by *Mtb* strains with poor *in vitro* autophagy-inducing ability displayed more severe radiographic extent of disease (Li et al., 2016). Consequently, inducing autophagy could limit lung damage.

## The Modulating Role of HIV

Globally, 13 percent of people with active TB who know their HIV status are co-infected with HIV-1 (World Health Organization, 2017). Although TB is also a risk factor for airflow obstruction in patients with HIV (Samperiz et al., 2014; Pefura-Yone et al., 2015; Gupte et al., 2017), in HIV positive patients with a low CD4 count (CD4 < 200/mm^3^) TB often presents with atypical CXR findings or even normal CXRs, while cavitation is 4-fold less common (Kwan and Ernst, 2011). These findings suggest that TB-related pulmonary damage might be reduced in HIV co-infected patients and the host immune response, necessary for protection against TB, is required for the development of cavities. Indeed, several of the factors previously discussed and implicated in pulmonary damage, are affected by HIV co-infection. For example, sputum levels of MMP-1, -2, -8, and -9 are reduced in HIV-TB co-infected patients, compared to patients without HIV (Walker et al., 2012, 2017) as is the activity and life span of neutrophils (Lowe et al., 2015). The effect of HIV co-infection on the levels of several of the other cytokines is variable across studies and thus it is difficult to interpret a clear trend (Zhang et al., 1994; Elliott et al., 1999; de Castro Cunha et al., 2005; Riou et al., 2012; Walker et al., 2012; Mihret et al., 2014; Kassa et al., 2016).

Paradoxical TB-associated immune reconstitution inflammatory syndrome (TB-IRIS) develops in approximately 18% (95% CI 16–21%) of patients on treatment for HIV-associated TB, usually within the first few weeks after starting ART (Namale et al., 2015). It results in new or recurrent TB signs and symptoms, commonly involving the lungs, such as cough, chest pain, and worsening radiographic pulmonary infiltrates. TB-IRIS is associated with increased levels of several cytokines, particularly IL-6, TNF-α and IFN-γ (Tadokera et al., 2011; Conesa-Botella et al., 2012; Lai et al., 2015; Ravimohan et al., 2015) and inflammasome activation (Lai et al., 2015). It results in increased neutrophil recruitment (Nakiwala et al., 2018), and up-regulation of MMP-1, -3, -7, -8, and -10 (Tadokera et al., 2014; Ravimohan et al., 2016; Walker et al., 2017). LT4AH also appears to play a role, as more severe TB-IRIS has been reported in patients with mutant (TT and CT) LTA4H genotypes (Narendran et al., 2016).

These findings suggest that TB-IRIS could result in pulmonary damage and impaired lung function. To date, only one study has explored the relationship between TB-IRIS and lung function in 14 patients with HIV-associated TB, 3 of whom developed TB-IRIS (Ravimohan et al., 2016). The study found that an increase in MMP-8 between baseline pre-ART and 4 weeks post-ART initiation strongly associated with impairment in lung function, but the small sample size limits definitive conclusions.

## Where Can We Intervene to Prevent or Reduce Lung Damage?

**FIGURE 2:**
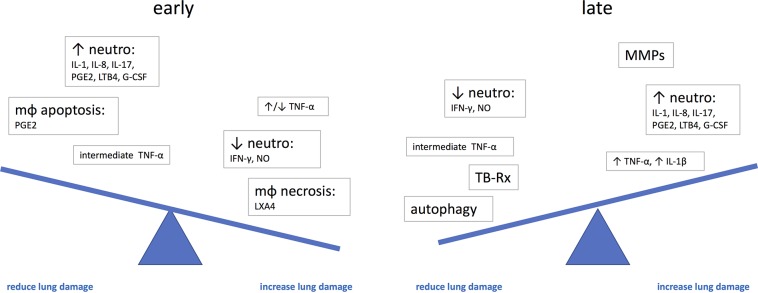
The potential effect of immune mediators on the development of lung damage at different stages of disease. G-CSF, granulocyte-colony stimulating factor; IL, interleukin; LTB4, leukotriene B4; LXA4, lipoxin A4; mφ, macrophage; neutro, neutrophils; NO, nitric oxide; PGE2, prostaglandin E2; TNF, tumor necrosis factor.

There are several uncertain areas around therapies to prevent or limit lung damage in TB. Changes in the lungs start to develop before clinical symptoms appear (Esmail et al., 2016; Zak et al., 2016; Scriba et al., 2017), and therefore, a large proportion of lung damage may already have occurred by the time the patient presents; several mediators of lung damage may have different roles at different stages of the disease; granulomas in various stages can be present at the same time in a single individual, and only a single or a few progressive granulomas can determine the outcome of the disease. Therefore, it remains uncertain what happens for example to the contained granulomas if we systemically treat the patient with potentially immunosuppressive therapy or what the right time is to intervene (Figure 2).

## Antituberculous Therapy as Host-Directed Therapy

Sputum *Mtb* load is associated with systemic inflammation and, combined with pre-treatment C-reactive protein levels, inversely correlates with CXR improvement 60 days after start of treatment (Mesquita et al., 2016). Time between first TB symptoms and start of treatment (de Valliere and Barker, 2004; Baez-Saldana et al., 2013), duration of treatment (Chung et al., 2011), and smear positivity (Chung et al., 2011) are associated with impaired pulmonary function, suggesting that prompt diagnosis and treatment will limit lung damage. In addition to a direct anti-mycobacterial effect, *in vitro* studies suggest that some antimycobacterial agents may have immunomodulatory action. Pyrazinamide directly reduces levels of TNF-α, IL-6 and IL-1β (Manca et al., 2013), quinolones downregulate MMP-1, -3, and -9 (Singh et al., 2014a), and rifampicin downregulates MMP-3 production by bronchial epithelial cells (Singh et al., 2014a) and inhibits PGE2 production (Yuhas et al., 2007). P-aminosalicylic acid (PAS), which is an aspirin derivate, suppresses PGE2-dependent MMP-1 production (Rand et al., 2009). Both isoniazid (INH) and pyrazinamide (PZA) enhance autophagy (Kim et al., 2012).

## Medicines Used in Other Human Diseases as Host-Directed Therapy for TB

In an adjunctive approach to TB therapy, treatment could be supplemented with host-directed therapies. Several readily available drugs affect cytokines, MMPs or eicosanoids and therefore potentially reduce pulmonary damage (Table 1).

**Table 1:** Host-directed therapies potentially inhibiting lung damage and/or promoting lung repair.

Host-directed inhibiting lung damage	Potential mechanism
Steroids	↓ INF-γ, TNF-α, IL-1β (and IL-6, IL-10, IL-12p40, and IP-10 in TB-IRIS)
	↓ MMP-7 (in TB-IRIS)
Doxycycline	↓ MMP-1, -3, and -9
Vitamin D	↓ MMP-7 and -9
	↓ IFN-γ, IL-6, IL-10, TNF-α
	↑ autophagy
Rapamycin, everolimus	↓ MMP-1 and -3
	↑ autophagy
NSAIDs	↓ PGE2^1^ and ↑ LXA4
Zileuton	↓ 5-LOX
Phosphodiesterase-4 inhibitors	↓ TNF-α
	↓ neutrophil recruitment
Metformin	↓ TNF-α
	↑ autophagy
Statins	↑ autophagy
TNF-α blockers	↓ TNF-α
PGE2	↑ PGE2^1^
IFN-γ	↑ IFN-γ
Mesenchymal stromal cells	Control inflammation and mediate tissue repair


*^*1*^The effect of inhibiting or increasing PGE2 on lung damage could vary depending on the stage of the disease.*

**Steroids** have been used as adjunctive treatment in TB for several decades (Dooley et al., 1997; Critchley et al., 2013), mainly in TB meningitis, pericarditis, and TB-IRIS, even though corticosteroid use without concomitant TB treatment increases the risk of developing TB (Jick et al., 2006). Two recent reviews concluded that there is no high quality evidence that steroid treatment significantly affects mortality or sputum conversion rate in pulmonary TB (Critchley et al., 2014; Schutz et al., 2018). An earlier review – including mostly studies done in the 1960s and patients not on rifampicin-based TB treatment – did find a beneficial effect of steroids on radiographic resolution and regression of cavities (Smego and Ahmed, 2003). A meta-regression analysis of 12 studies found steroids do accelerate sputum TB culture conversion (Wallis, 2014) – which is inversely associated with development of airflow obstruction (Radovic et al., 2016); however, high doses (134 mg prednisone daily) for an extended period (2 months) are required to reach clinically relevant outcomes (Wallis, 2014). Moreover, the only two studies in this analysis in which patients were on rifampicin-based treatment show contradicting results.

Corticosteroids inhibit various cytokines in TB (IFN-γ, TNF-α, IL-1β) and TB-IRIS (IL-6, IL-10, IL-12p40, TNF-α, IFN-γ, and IP-10) (Mahuad et al., 2004; Mayanja-Kizza et al., 2005; Meintjes et al., 2012; Bongiovanni et al., 2015). In patients with tuberculous meningitis, the effect of corticosteroids was found to be LTA4H genotype modulated, with only patients with the mutant TT genotype, leading to a higher inflammatory response, benefitting from steroid treatment (Tobin et al., 2012). In patients with TB-IRIS, however, this difference in genotype on the effect of steroid treatment was not confirmed (Narendran et al., 2016). The effect of corticosteroid treatment and TB-IRIS on pulmonary function is being assessed in a substudy of the PredART trial (Meintjes et al., 2017).

Little evidence is available for other **TNF-α blocking therapies**. A trial of 16 patients with HIV-associated TB treated with etanercept (but no ART) showed a tendency to greater CXR improvement from baseline to 6 months compared to a placebo group, although this was not statistically significant (*p* = 0.2) (Wallis et al., 2004). Case reports describe successful treatment of paradoxical TB reactions or TB-IRIS – involving the pleura, lymph nodes or brain – with infliximab (Blackmore et al., 2008; Jorge et al., 2012; Hsu et al., 2016), or adalimumab (Wallis et al., 2009; Lee et al., 2012). Although only one case refers to pulmonary TB-IRIS [occurring after interruption of prior anti-TNF-α treatment (Wallis et al., 2009)], these case reports support the possible benefits of TNF-α blockers in the treatment of (complicated) TB. Restarting TNF-α blockers during or after TB treatment was safe and only led to one recurrence of TB in a cohort of 22 patients in Turkey followed for a median of 53 months (Ozguler et al., 2016).

Doxycycline is the only licensed **MMP-inhibitor** for use in humans. It suppresses MMP-1, -3, and -9 secretion by *Mtb* infected human macrophages and bronchial epithelial cells (Walker et al., 2012). Other agents also inhibit MMPs *in vitro*: prednisone – in patients with TB-IRIS – suppresses MMP-7 gene expression (Tadokera et al., 2014), vitamin D inhibits secretion of MMP-7 and -9 (Anand and Selvaraj, 2009; Coussens et al., 2009), and **rapamycin** (an mTOR-inhibitor and a known autophagy inducer that can also affect macrophage polarization (Mercalli et al., 2013)) inhibits MMP-1 and MMP-3 (Singh et al., 2014b). Use of the latter in TB is limited by the interaction with rifampicin. In mice, broad spectrum inhibition of MMPs enhances the efficacy of INH and RIF treatment (Xu et al., 2018). Conceptually, inhibition of MMPs may lead to less pulmonary damage, but so far, no clinical trials have directly assessed this. Currently, everolimus, a rapamycin derivate, is being tested as HDT in patients with moderate to far advanced pulmonary tuberculosis (together with vitamin D, auranofin [a gold complex with antimicrobial activity used in rheumatoid arthiritis], and CC-11050 [a phosphodiesterase 4 (PDE4) inhibitor]), using rifabutin-based anti-TB treatment (ClinicalTrials.gov NCT02968927); with change in FEV1 being one of the secondary outcomes. Both rapamycin, its derivates, and vitamin D could theoretically reduce lung damage through inhibition of MMPs, although the effect of vitamin D treatment on CXR abnormalities is variable (see below). PDE4 inhibitors, in combination with INH treatment, have been shown to reduce TB-associated lung damage in rabbits (Subbian et al., 2011) and pulmonary bacillary load in mice (Maiga et al., 2015). Doxycycline is being investigated for its potentially modulating effect on tissue destruction in pulmonary TB (ClinicalTrials.gov NCT02774993).

**NSAIDs** inhibit the enzyme cyclooxygenase (COX), thereby inhibiting PGE2 production and enhancing LXA4 production. An adjunctive role for NSAIDs in treatment of human TB has only been shown for acetylsalicylic acid in reducing PZA-induced arthralgia (Petty and Dalrymple, 1964; Horsfall et al., 1979) and possibly in TB meningitis (Misra et al., 2010; Schoeman et al., 2011; Mai et al., 2018). Negative effects have been described: a Taiwanese study found an association between NSAID use (both traditional NSAIDs and selective COX-2 inhibitors) and an increased risk of active TB (Wu et al., 2017). However, it is not clear whether this association is causative (i.e., decreased apoptosis at the very early stages of TB) or merely reflects an increased use of NSAIDs early during TB. In mice, inhibition of PGE2 by the NSAID ibuprofen was shown to affect lung pathology: inhibition early in the disease process leads to an increase in pulmonary inflammation and pathology (Rangel Moreno et al., 2002), whereas inhibition later during disease decreased lung pathology and neutrophil influx (Rangel Moreno et al., 2002; Vilaplana et al., 2013). Increasing **PGE2** by early (day one post infection) administration of exogenous PGE2 (dinoproston – normally used for induction of labor) and/or the 5-lipo-oxygenase inhibitor zileuton (used in the treatment of asthma) to IL-1 deficient mice resulted in less necrotic lung pathology by TB (Mayer-Barber et al., 2014). No studies with dinoproston or zileuton have been performed in human TB to date. A pilot study is currently investigating the effect of ibuprofen added to multi-drug resistant TB treatment on radiological improvement of TB, amongst other endpoints (ClinicalTrials.gov NCT02781909).

In *in vitro* models, **metformin,** a widely used antidiabetic agent, has been shown to inhibit TNF production by monocytes (Arai et al., 2010), affect macrophage polarization (Nadella et al., 2017), and promote autophagy (Singhal et al., 2014). It affects Th1 responses, but data are conflicting: in mice infected with TB, metformin treatment promotes the expansion of *Mtb*-specific IFN-γ secreting T cells in the lungs (Singhal et al., 2014), whereas in human THP-1 cells (not infected with *Mtb*) metformin suppressed the production of Th1-related cytokines (Chen et al., 2018). Metformin use in patients with diabetes mellitus on treatment for TB was associated with decreased mortality compared to patients using other anti-diabetic drugs in two retrospective observational cohorts (Singhal et al., 2014; Degner et al., 2018). A retrospective cohort study of TB patients with diabetes mellitus showed that those using metformin at diagnosis and during TB treatment had fewer cavities and fewer CXR abnormalities compared to those using other anti-diabetic drugs (Singhal et al., 2014). Another retrospective study, however, showed increased cavitatory disease in patients using metformin (Degner et al., 2018).

**Vitamin D3** induces autophagy (Campbell and Spector, 2012) and inhibits the secretion of MMP-7, -9 (Anand and Selvaraj, 2009; Coussens et al., 2009), and several cytokines, for example IFN-y, TNF-α, IL-6, and IL-10 (Vidyarani et al., 2007; Harishankar et al., 2014) *in vitro*. However, its effect on radiological outcomes are ambiguous: three trials comparing vitamin D3 as adjunctive therapy demonstrated no effect on CXR score (Martineau et al., 2011; Ralph et al., 2013b; Mily et al., 2015) or pulmonary function (Ralph et al., 2013b), while one study found more CXR improvement in the vitamin-D3 treated group (Salahuddin et al., 2013).

**Statins** are widely used inhibitors of cholesterol biosynthesis. They induce autophagy *in vitro* (Parihar et al., 2014) with broad anti-inflammatory effects, although not directly demonstrated in TB (Hennessy et al., 2016). Their use has been associated with a reduced risk of developing active TB in some studies (Lai et al., 2016; Liao et al., 2017; Su et al., 2017), but not in all (Kang et al., 2014). No studies have been performed in humans assessing statins in relation to pulmonary damage in TB; in mice, statins have been found to reduce lung pathology (Parihar et al., 2014). A future study will look at the effect of pravastatin added to standard TB treatment on pulmonary function (NCT03456102).

Several studies looked at the effect of **IFN-γ** as adjunctive therapy for TB (Gao et al., 2011). The studies were small, and most were performed in patients with multi-drug resistant TB. Aerosolized IFN-γ in combination with TB treatment resulted in better CXR outcomes compared to TB treatment alone. This contradicts the finding in mice, where adding IFN-γ resulted in worse pulmonary outcomes (Sakai et al., 2016). The authors conclude that IFN-γ might be beneficial as adjunctive therapy in TB, but larger trials are needed to confirm this.

**Mesenchymal stromal cells** are tissue-resident non-hematopoietic adult progenitor cells. They are believed to facilitate organ homeostasis and tissue repair and can modulate immune responses; they have been used in treatment of graft-versus-host-disease and autoimmune diseases (Parida et al., 2015). In a phase 1 trial in patients with drug resistant TB, infusions of autologous mesenchymal stromal cells, 4 weeks after starting TB treatment, was safe and resulted in CXR improvement in 25/36 patients compared to 15/36 controls (Skrahin et al., 2016).

## Conclusion

The immune mechanisms of parenchymal lung damage in human TB are complex and incompletely understood. The difference between pulmonary damage in animal models (mostly occurring as a result of primary TB) and humans (mostly occurring as a result of post-primary TB) further complicates study of this phenomenon. Processes taking place in the lung are heterogeneous, with granulomas with varying degrees of mycobacterial control existing next to each other and inflammatory cells and cytokines appearing to have different effects at different time points. MMPs seem to play an important role and consequently, inhibition of MMPs may lead to reduction in pulmonary damage, however, this remains to be proven in clinical trials. Neutrophils are another key mediator of pulmonary damage, whose recruitment could potentially be inhibited by NSAIDs. The role of other effectors is less clear and better insight into their effects over the course of TB infection and disease is needed to be able to guide potential intervention. Future studies of human TB and (host-directed) therapy should include radiographically assessed lung damage and pulmonary function as an outcome.

## Author Contributions

CS wrote the first draft of the manuscript. GM, BA, NW, RW, and LL critically revised the manuscript. All authors read and approved the submitted version.

## Conflict of Interest Statement

The authors declare that the research was conducted in the absence of any commercial or financial relationships that could be construed as a potential conflict of interest.
